# Effects of Roughage Quality and Particle Size on Rumen Parameters and Fatty Acid Profiles of Longissimus Dorsi Fat of Lambs Fed Complete Feed

**DOI:** 10.3390/ani10112182

**Published:** 2020-11-22

**Authors:** Abdulkareem M. Matar, Mutassim M. Abdelrahman, Ibrahim A. Alhidary, Moez A. Ayadi, Mohsen M. Alobre, Riyadh S. Aljumaah

**Affiliations:** 1 Department of Animal Production, Faculty of Food and Agriculture, King Saud University, P.O. Box 2460, Riyadh 11451, Saudi Arabia; Abdmatar@ksu.edu.sa (A.M.M.); ialhidary@ksu.edu.sa (I.A.A.); moez_ayadi2@yahoo.fr (M.A.A.); malobre@ksu.edu.sa (M.M.A.); rjumaah@ksu.edu.sa (R.S.A.); 2 Département de Biotechnology Animal, Institute Superior de Biotechnology de Beja, University de Jendouba, B.P. 382, Av. Habib Bourguiba, Beja 9000, Tunisia

**Keywords:** fatty acids, roughage and particle size, lambs, longissimus dorsi

## Abstract

**Simple Summary:**

Roughage type and particle size may play an important role in rumen volatile fatty acid levels and consequently affect fatty acid profile in meat and quality. Two roughage sources and two particles were used in this trial for growing lambs to study the effect in volatile fatty acids levels and fatty acids profile of longissimus dorsi (LD) fat. The results of this trial supported the hypothesis by confirming the effect of roughage type and particle size on meat quality and human health.

**Abstract:**

The fatty acid composition for the longissimus dorsi (LD) fat of carcass sheep is a crucial factor impacting meat quality. We performed a 90-day feeding trial of 25 Naemi lambs to investigate the effects of roughage sources (alfalfa or wheat straw) of two sizes (regular and 1 cm chopped) when fed with pelleted total mixed ration (TMR) on the growth performance, fermentation patterns, and fatty acid (FA) composition of longissimus dorsi (LD) fat. Lambs were randomly assigned to individual pens with five treatment diets, as follows: C, control group with TMR; T1, TMR and regular alfalfa hay; T2, TMR and alfalfa hay chopped to 1 cm; T3, TMR and regular wheat straw; and T4, TMR and wheat straw chopped to 1 cm. Four lambs were randomly selected from each treatment (20 total) and sacrificed. LD fat of the carcass was extracted and analyzed for FA using a gas chromatography-mass spectrometry. Significantly increased feed intake was found in T1 and T2. The FA composition of LD fat in T2 had higher unsaturated fatty acid (UFA), omega-6 (n6), and omega-3 (n3) FA content. Conjugated linoleic acid (CLA) and α-linoleic acid were highest in lambs fed T1 and T2. Feeding different types of roughage, especially alfalfa hay, either regular or chopped, with total pelleted mixed ration is crucial to improving feed intake and body weight gain, as it positively enhances the rumen microbial fermentation process by controlling rumen pH. The FA profiles of meat from lambs fed TMR with regular or 1 cm particle size alfalfa hay (T1 and T2) are recommended for human consumption as a source of healthy FAs.

## 1. Introduction

Human nutritionists support the idea that improving the nutritional balance of foods, including meat, is the best way to provide the well-balanced nutrients in the diet as a whole [1]. In recent years, it has become clear that the fatty acid (FA) composition of meat can be changed by altering dietary content during animal production so that the meat more closely meets nutritional guidelines. This applies to ruminant species, as well as to pork and chicken [2]. The composition of FAs in fat deposits of sheep has received little research interest, compared to milk and other meat animals [3,4]. The characteristics of the food consumed play an essential role in maintaining health and preventing human disease. There has traditionally been considerable consumption of mutton, but in the last decade, this has decreased significantly. Among the reasons for this decrease is the association of consumption of red meat content highly saturated fatty acid (SFA) with the development of cardiovascular diseases and increasing concern for a healthier diet that is low in saturated fatty acids (SFAs) [5] despite the known nutritional benefits of meat as it provides high-quality protein and important minerals, including iron and zinc [6]. It has been rumored in our Arab society that meat fat causes many serious health problems, such as arterial blockage, high cholesterol, and heart attacks, which has led many people to refrain from consuming it—but so far, there are no studies confirming the opposite. The composition of FA profiles of lipids present in meat has received increasing attention, due to its implications for product quality and human health. The proportions of polyunsaturated fatty acid (PUFA), omega-6 (n-6), and omega-3 (n-3 PUFA) are widely used to evaluate the nutritional value of food. Recently, most studies have focused on the impact of individual FAs, such as conjugated linoleic acid (CLA) and the ratio of CLA/SFA on the prevention of cardiovascular disease and anticancer activity [2,7]. The lipids in meat and milk are mainly composed of triacylglycerols containing more than 400 different FAs [8]. This fat diversity is due to the lipid metabolism during the microbial rumen fermentation process and the enzymatic activity of the mammary gland in milk [9]. The process of bio-hydrogenation (BH) in the rumen by microorganisms is the main process that controls the FA profiles of the milk and tissues of ruminants. Along with the transformation of unsaturated fatty acids (UFAs) in feed into several saturated forms, BH is responsible for the synthesis of numerous biohydrogenation intermediates (BHI), such as geometric isomers of monoenes and dienes, which can be deposited in meat [10]. Dietary type, composition, and particle size are the main factors that determine fat microbial fermentation and volatile fatty acid (VFA) production levels, which affect the FA composition of ruminant end products [11,12]. The forage feeding value is mainly determined by feed intake, saliva production, rumination, and the passage rate of feed in the rumen, which primarily depends on particle size [13]. Forage particle size also influences BH pathways and FA composition in lamb meat [14]. Recently, the traditional production system for growing lambs has shifted to an intensive feeding system, using complete feed as a total mixed ration (TMR). Sheep feeding systems show great variability in results, which is related to a large number of sheep breeds and the variation in forage production and quality. On the other hand, some secondary plant compounds can improve production parameters owing to their effects on digestibility, thereby reducing energy losses, due to reduced methane production [15]. We hypothesized that forage quality and particle size has an effect on the FA composition in fat deposits to improve the meat quality of Naemi lambs and become healthier for human. The objective of this study was to evaluate the effect of the particle size of alfalfa and wheat straw fed with TMR on feed intake, rumen fermentation process, and FA composition of the longissimus dorsi (LD) fat of Naemi lambs.

## 2. Material and Methods

### 2.1. Animals, Diets, and Management

The trial was conducted at the experimental farm (Alamaaria), Department of Animal Production, King Saud University, Riyadh, Saudi Arabia. Animal care and management procedures were performed in strict adherence to the guidelines of the Saudi Arabia Regulations for the Use and Care of Animals in Research, King Saud University, approval # KSU-SE-20-56. Twenty-five weaned Naemi lambs were purchased from a local market. They had an average body weight of 28.7 ± 1.3 kg. Lambs were randomly assigned to individual pens (1.5 m long × 1.0 m wide) under the same environmental conditions with permanent access to clean water. Five treatment diets were prepared, as follows: C, control group fed total mixed ration (TMR); T1, TMR and regular alfalfa hay; T2, TMR and alfalfa hay chopped to 1 cm particle size; T3, TMR and regular wheat straw; and T4, TMR and wheat straw chopped to 1 cm particle size. The ingredients, chemical composition, and FA profiles for all experimental diets are reported in Table 1. The TMR ingredients were formulated according to [16] to cover the daily nutrient requirements of the growing lambs. The adaptation period was 14 d before starting the trial, and the lambs were adapted to the pens and the primary diet. All lambs were offered the diets at 8:00 a.m. every day at 3.0% *ad libitum*, which was weekly calculated according to body weight changes of the growing lambs.

### 2.2. Slaughtering and Sample Collection

At the end of the feeding trial, four lambs were randomly selected from each treatment (20 lambs) and sacrificed after 12 h fasting. Lambs were slaughtered at the Dir’aiyah abattoir, located about 17 km from the lambs’ stalls. The slaughtering was performed in an appropriate ritual manner—the lamb’s throat was cut by a sharp knife severing the carotid artery and jugular vein, and the blood was drained from the carcass. A proper process was followed, and care was given to reduce fear and stress during transport and slaughter, to avoid the negative effects of stress on meat quality. The live weight of lambs was recorded, and immediately after slaughter, the carcass weight was recorded. Carcasses were kept at 4 °C for 24 h to prevent cold shortening. All carcasses were divided into right and left sides. The longissimus dorsi (LD) muscle of the right side of the carcass was extracted. One steak fat, approximately 20–30 mm thick, was cut from the region of the 7th rib of the LD muscle for fatty acid analysis. Chops were plastic-packed and stored at −20 °C until analysis to avoid lipid oxidation. In addition, feed samples were analyzed for FA composition. The feed offered and refusals were weighed weekly, and the feed intake was calculated on a DM basis. Samples of rumen fluid (50 mL) were collected from each lamb at the end of the trial (90 d) by using an oral stomach tube connected to a pump and then immediately stored frozen at −20 °C to determine the VFA level using gas chromatography.

### 2.3. Fatty Acid Composition Analysis of Lipid Deposits

The process of methylation and extraction for each of the sample fat LD muscle was performed, while it was still frozen. The sample was first sliced (1 mm maximum) and then prepared according to the method proposed by Vasta et al. [17] for meat. The analysis of all FA methyl esters (FAMEs) was performed according to the method reported by Matar et al. [18]. Briefly, FAMEs were obtained using a gas chromatography-mass spectrometry ultra-instrument (GCMS-QP2010, Shimadzu, Kyoto, Japan) and a Rtx-1 column (30 m × 0.25 mm i.d., 0.25 μm film thickness), with helium as the carrier gas at a flow rate of 1.41 mL min^−1^. The temperatures of the injector and detector were 220 °C and 275 °C, respectively. For gas chromatography-mass spectrometry detection, an electron ionization system with ionization energy of 70 eV was used. The ion source temperature was 230 °C, and the interface temperature was 280 °C. The individual FA was identified and expressed as a percentage of the total amount of FAMEs identified based on the ratios of the peak area of the FA species to the total peak area. The sums of SFAs, UFAs, PUFAs, n-6, n-3, and the n-6:n-3 ratio were collected.

Quantified VFAs in ruminal fluids were determined by using a gas chromatography-mass spectrometry ultra-instrument (GCMS-QP2010, Shimadzu, Kyoto, Japan) and an Rtx-1 column (30 m × 0.25 mm i.d., 0.25 μm film thickness) with helium as the carrier gas at a flow rate of 1.50 mL min^−1^. The injector temperature was 250 °C, and the detector temperature was 280 °C. VFAs were quantified using calibration curves, which were prepared for each VFA at concentrations ranging from 0.2 to 30 mmol/L and using C5:0 as an internal standard at 50 mmol/L, according to the methodology described by Reference [19].

### 2.4. Statistical Analysis

The data for the performance and FA composition of LD fat were analyzed in a randomized complete block design using the Proc GLM procedure of SAS 9.4 (SAS Institute Inc., Cary, NC, USA). The following model was assumed:

yij = μ + τi + βj + εij,(1)

where yij = an observation of performance and fatty acid composition of diet I; fat muscle in lambs j; μ = the overall mean; τi = the effect with treatment i included: C, including, total mixed ration (TMR); T1, including TMR and regular alfalfa hay; T2, including TMR and alfalfa hay with 1 cm particle size; T3, including TMR and regular wheat straw; and T4, including TMR and wheat straw with 1 cm particle size; βj = the animal effect of block j; and εij = residual error. Data are presented as the mean μ and SEM, and differences were considered significant at *p* < 0.05.

## 3. Results

### 3.1. Growth and Feed Efficiency of the Growing Lambs

Table 2 presents the effect of feeding by alfalfa hay and wheat straw with different particle sizes on the growth performance of Naemi lambs fed complete feed. The lambs did not show any effect on animal performance, so the β_j_ factor effect wasn’t discussed l. Significantly higher feed intake was reported for lambs from T1 and T2 compared with the other groups. The same trend was reported for total body gain, which was numerically, but not significantly different from that of the other three groups. A lower feed conversion ratio (FCR) was reported for lambs from T1, T2, and T4 than from C and T3.

### 3.2. Rumen Volatile Fatty Acids

The effects of the quality of dietary roughage and particle size on ruminal VFA content are presented in Table 3. The results for chopped alfalfa (T2) and regular and chopped straw (T3 and T4, respectively) were significantly higher in acetic acid (AA) compared to the other treatment groups. In contrast, butyric acid (BA) was higher in the control and treatment with regular and chopped alfalfa hay in T1 and T2. Propionic acid (PA) was significantly higher (*p* < 0.05) in the control (TMR) compared to the other treatment groups. Furthermore, a significantly higher AA:PA ratio was reported for lambs fed TMR with chopped alfalfa hay (T2) compared with all other dietary groups. Significantly lower values were found for lambs from the control group (C; 1.15), but no significant differences were reported among the other groups (T1, T2, and T3).

### 3.3. Fatty Acid Profiles of LD Muscle Fat

The FAs found in animal tissues and products reflect the processes that take place in the rumen, particularly the ruminal biohydrogenation process [20,21]. The FA composition of the LD muscle fat in this study mainly consisted of oleic (C18:1), palmitic (C16:0), and stearic (C18:0) acids. The proportions of small and mid-chain fatty acids, such as capric acid (C10:0), lauric acid (C12:0), and myristic acid (C14:0) were not affected (*p* > 0.05) by the treatments. Notably, no significant variations in the proportions of myristic acid C14:0, palmitic acid C16:0, and stearic acid C18:0 were found among all treatments. This result was in agreement with the study by Zhang et al. [22] on Dorper, Tan, and Hu lambs.

The analyzed FA composition of LD fat, as expected, showed that a diet, including alfalfa hay with particle sizes T2 and T3 had a higher content of palmitoleic acid C16:1 cis9, while T2 had higher significantly increased content of FAs, including: oleic acid C18:1 cis9 and α-linoleic acid C18:3n-3, as shown in Table 4. At the same time, diets, including alfalfa hay and wheat straw (T2 and T4), had a marked increase in linolenic acid, C18:2n-6 (*p* < 0.01). Conjugated fatty acid (CLA) C18:2 *cis9 trans11* was numerically increased in T2 by 48% compared to the control dietary treatment. On the other hand, the C diet with TMR was highly effective for nonadecanoic acid, C19:0 (*p* < 0.01) as compared to the other treatment groups. In Table 5, most changes in FA proportions were found in lambs with roughage in their diets. The proportion of SFA was reduced in all diets with roughage compared to C when TMR was used. A similarly increased proportion of UFA, ∑n3 (*p* < 0.02), Δ9C18 (71.04%), and ∑hFA (*p* < 0.02) was noted in T2 with alfalfa and 1 cm particle size. Likewise, the lambs from T4, wheat straw with 1 cm particle size, had significant variations in the proportions of PUFA and ∑n6 ((*p* < 0.03; *p* < 0.04, respectively) of the LD muscle, as shown in Table 5. In contrast, the AI was less valuable (0.95%) for lambs from T2 compared to other treatments.

## 4. Discussion

### 4.1. Growth and Feed Efficiency of the Growing Lambs

Feed intake and efficiency are two factors that determine animal growth performance. The high feed intake observed in diets supplemented with alfalfa, both regular and chopped, compared to those with wheat straw and TMR, was reflected in the high significant total body gain and lower feed conversion of the lambs (Table 2). This result could be explained by the high nutritive values of alfalfa in terms of protein compared with wheat straw [16]. Our results were similar to those of Santos-Silva et al. [23]. The results of our trial disagreed with other reports on Merino Branco lambs reared under similar conditions and with similar carcass weights [14]. The body weight gain differed between the dietary groups, but the meat quality and FA profiles varied as a result of variation in the microbial fermentation process.

### 4.2. Rumen Volatile Fatty Acids

Lambs fed diets of chopped alfalfa (T2), and regular and chopped straw (T3 and T4, respectively) showed significantly higher AA compared to other treatment groups. As a general trend, a higher level of AA in these groups is related to their high fiber and forage intake, which increases saliva secretion through rumination and consequently maintains rumen pH [24]. Previous studies [25,26] have reported higher AA levels for steers fed a higher level of forage, which agreed with our findings. In contrast, BA was higher in the control and treatment with regular and chopped alfalfa hay, T1, and T2. PA was significantly higher (*p* < 0.05) in the control (TMR) compared to the other treatment groups because of the high intake of concentrate, which may cause a reduction in rumen pH and consequently lower rumen AA and increased PA. Likewise, a significantly higher (*p* < 0.05) AA:PA ratio was reported for lambs fed TMR with chopped alfalfa hay (T2) compared to all other dietary groups, as shown in Table 3. Significantly (*p* < 0.05) lower values were found for lambs from the control group (C; 1.09), but no significant differences were reported among other groups (T1, T3, and T4). The VFA levels for all groups differed because of the dietary levels of fiber consumed and the feed intake, which differed from one group to another. This variation plays an important role in the differences in FA accumulation in the tissues, especially LD, as reported in the following tables.

### 4.3. Fatty Acid Profile of LD Fat

With respect to other species, sheep fat is similar to that of other ruminants, such as cattle [2]. The fat of ruminant animal deposits is predominantly saturated and monounsaturated, with palmitic (C16:0), stearic (C18:0), and oleic (C18:1 c9) acids representing around 80% of the total FAs. In this trial, the FA composition in LD muscle fat was similar in all treatments when roughage was included. These results were desirable, as an increase in muscle fat is generally accompanied by a reduction in SFA proportions and an increase in PUFA and MUFA proportions in LD muscle fat.

The LD muscle fat of Naemi lambs shows high amounts of palmitic acid and is positively correlated with high levels of blood cholesterol; this may be a response to reduced activity of the low-density lipoprotein (LDL) receptor [27]. Increased levels of high-density lipoprotein (HDL) in the plasma depend on oleic acid and stearic acid, which play a role in the hypocholesterolemic function and absorb cholesterol crystals [28].

Oleic acid, for which numerous beneficial effects on human health are known, represents the majority of all fatty deposits in proportions of around 35–40% in muscle fat, followed by palmitoleic acid [29]. Notably, the proportion of oleic acid was lowest in the fat muscle of Naemi lambs fed the TMR diet. However, the level of oleic acid intake was higher in TMR, as illustrated in Table 1. This could be due to the lower uptake from the blood and/or lower synthesis from stearic acid by the action of D9C18 desaturase, as suggested by the lower desaturase indices (Δ9C18) [28]. The increased and decreased content of oleic acid and palmitic acid in animal fat deposits may result from the increased conversion of palmitic acid into oleic acid across elongation and desaturation [28]. In this study, the oleic acid C18:1n-9 contents were lower in Neami lamb 35% compared to Dorper, Tan, and Hu lambs had a higher proportion (40.72, 40.37, and 40.60, respectively) [22].The disagreement results with the previous studies may be returned in the first instance on the genotype of lambs, which is related to rumen metabolism and metabolic rate [30].

PUFAs, which include the n-6 and n-3 families, are the most important, and the critical FAs within each family are linoleic acid n-6 (LA) and α-linolenic acid n-3 (ALA); that is, they cannot be synthesized in the body and must be obtained from the diet [31]. ALA is essential for biological actions involving gene expression, cell membrane integrity, and lipid mediators, and also plays an important role in the prevention of cardiovascular disease, inflammatory diseases, metabolic diseases, and cancer [32]. On the other hand about meat flavor the evidence approves there is a link between reducing the mutton-like odor intensity and LA, while ALA increases unpleasant odor in lamb meat [33].

**Figure 1:**
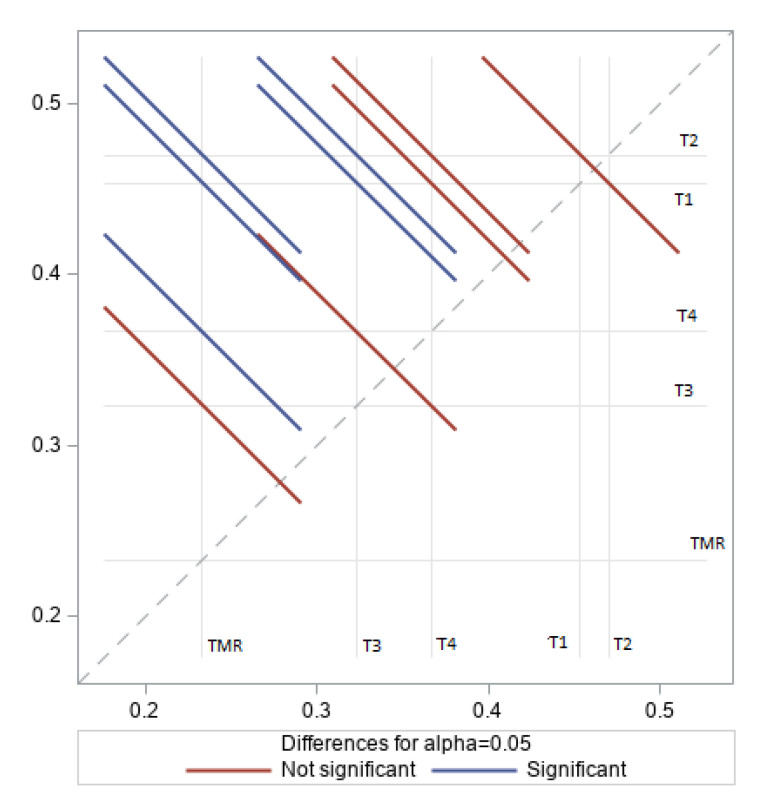
Effect of treatments on α-linoleic acid (ALA %) content of longissimus dorsi (LD) fat muscle in Naemi lambs.

**Figure 2:**
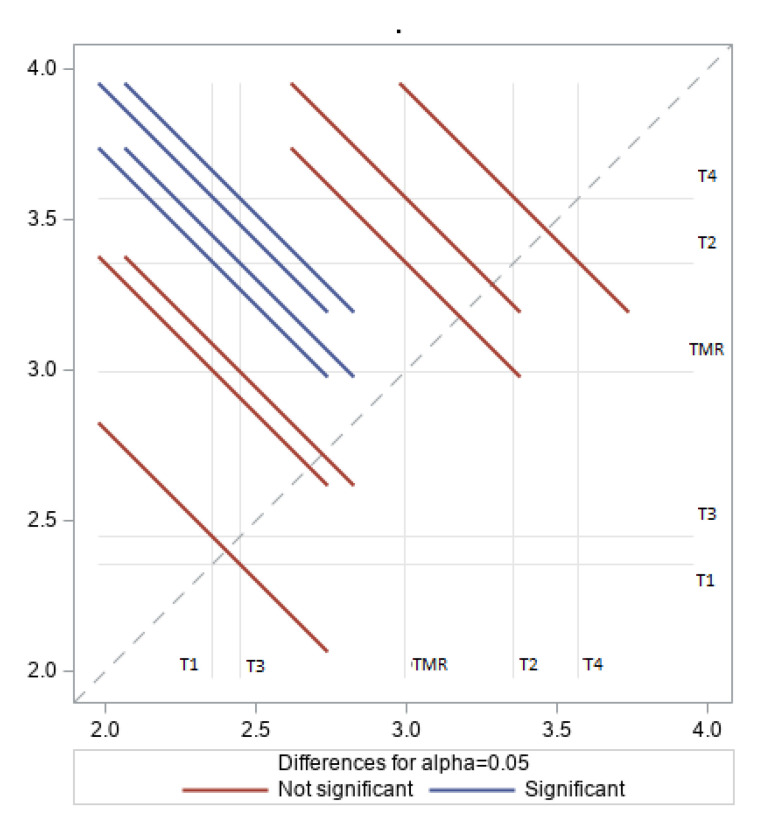
Effect of treatments on linoleic acid (LA %) content of longissimus dorsi (LD) fat muscle in Naemi lambs.

In this study, lambs fed TMR had lower α-linoleic acid in the LD muscle fat compared to lambs raised on roughage diets, as illustrated in Figure 1. This is probably because forage diets (TMR) are rich in α-linoleic acid. Additionally, since α-linoleic acid is degraded in the rumen, the large amounts of forage consumed by ruminants mean that a significant amount is absorbed. Chikunya et al. [34] found that 91% of α-linoleic acid was biohydrogenated in sheep, leaving 9% for absorption. A study on [35] lambs fed protected linseed and soybean diet showed a double percentage of C18:3 n-3 in the muscle lipids (3.8%) compared to other linseed oil diets. In the same context, feeding TMR had no effect on linolenic acid C18:2 n−6 proportions in the LD fat muscle, as illustrated in Figure 2, despite the high LA content of the diet. Similar results were reported for lambs fed TMR supplemented with 9% linseed; these displayed less linolenic acid in the muscle fat compared to lambs raised on pasture or high-roughage diets [36,37,38].

CLA plays a vital and important role because of its potent anti-obesity, anti-inflammatory, and anti-carcinogenic activity and immunomodulatory effects [39]. In this experiment, the LD muscle fat content of *cis 9*, *trans11*, C18:2 (CLA) was highest in lambs fed roughage (alfalfa), with decreased content found in TMR treatment. The proportion of CLA can be affected by impaired biohydrogenation of C18:2n−6 in the rumen, or across increased production of vaccine acid *trans*,*11* C18:1, and CLA can be produced in the tissues [40].

Arachidonic acid (C20:4n-6) is considered an essential FA that plays a role in reducing the risk of thrombosis [41], and meat is an important source of arachidonic acid. Notably, the proportion of C20:4n-6 in our study did not differ between treatments. This indicates that arachidonic acid synthesis occurs across the ruminal processes of elongation and desaturation of the intermediate products of biohydrogenation [42].

SFA in ruminant meat can originate from the diet, and are synthesized from dietary UFA in the rumen or from acetate and glucose in the liver or adipose tissue [43]. The high level of total SFAs in lambs fed TMR diet contrasted with the lower content of SFAs and a higher proportion of UFAs in T1and T2. French et al. [44] reported that the proportion of concentrate in the ration could be decreased effectively, leading to an increase in the PUFA: SFA ratio and a decrease in SFA in the muscle fat. Excessive SFA in the diet plays a role in promoting adipose tissue amplification and leads to the release of inflammatory proteins, such as cytokines and chemokines, which increase insulin resistance and induce inflammation, thereby increasing the risk for metabolic syndrome and cardiovascular disease [45].

**Figure 3:**
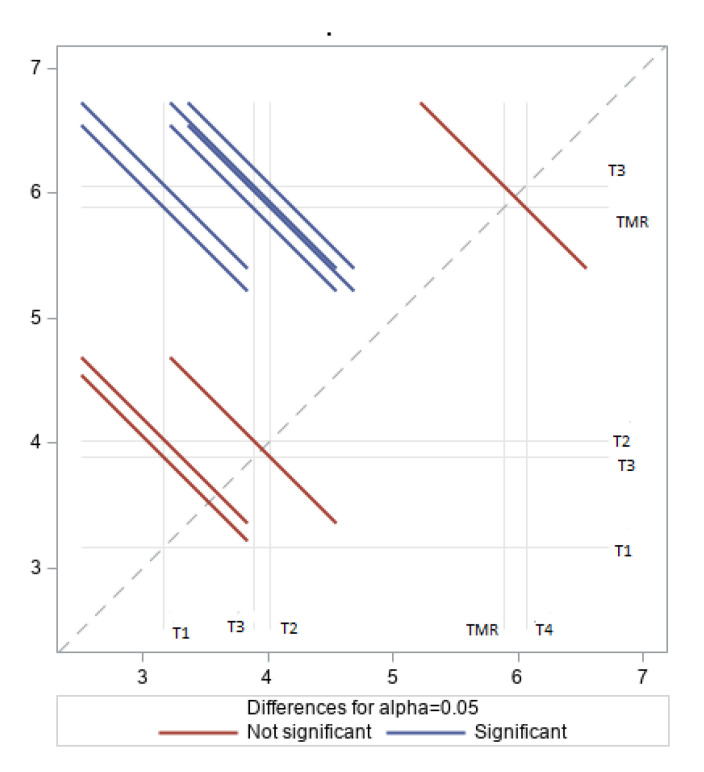
Effect of treatments on n-6/n-3 % content of longissimus dorsi (LD) fat muscle in Naemi lambs.

A higher proportion of PUFA in the LD fat was found in lambs fed wheat straw and alfalfa (T4 and T2). Ruminants fed forage have meat fat higher in n-3 PUFA content compared to that of ruminants raised on concentrate-based diets [38,46]. The n-6/n-3 ratios below 4.0 in the LD fat of lambs fed alfalfa (T1) and wheat straw (T3) are illustrated in Figure 3. The n-6/n-3 ratios in LD fat for all diets were between those of the PUFA series and recommended guidelines [47]. A previous study [48] indicated that lambs fed with concentrate presented n-6/n-3 values of 8.4 in muscle fat, while grazing lambs supplemented with concentrate and castrated lambs on pasture showed values of 2.5 and 1.1, respectively.

The atherogenic (AI) and thrombogenic (TI) parameters are considered important factors that highlight the UFAs to avoid excessive intake of saturated lipids. The AI value in our study was lower (0.95) in T2 with 1 cm alfalfa than in other treatments. This value for AI was similar to that found by Madruga et al. [49] and lower than that reported by Costa et al. [50] in Santa Inês lamb meat. Thus, the meat fat of the animals fed TMR and alfalfa is considered ideal for human consumption owing to its health benefits, such as the prevention of atherogenic and thrombogenic effects.

## 5. Conclusions

Feeding different types of roughage, particularly regular or chopped alfalfa hay, with total pelleted mixed ration (TMR) is crucial for improving feed intake and body weight gain, probably by positively enhances the rumen microbial fermentation process and consequently affecting rumen pH. The parameters for fatty acid profiles of meat from lambs fed TMR with regular or 1 cm particle size alfalfa hay (T1 and T2) are recommended for human consumption as a source of healthy FAs compared with meat from lambs fed wheat straw.

## Figures and Tables

**Table 1:** Chemical composition and fatty acid profiles of the experimental diet.

Item	Alfalfa Hay	Wheat Straw	TMR
Chemical Composition (Dry Matter Basis)			
Dry matter	90.4	90.3	90.14
Crude fiber	24.1	41.6	11.98
Crude protein	16.2	3.90	12.8
Neutral detergent fiber (NDF)	45.2	18.0	41.95
Acid detergent fiber (ADF)	32.9	50.0	26.10
Ether extract	2.50	1.40	2.75
ME, Mcal/kg ^1^	2.10	1.50	2.86
Fatty Acids Composition (% by weight)			
C8:0	7.82	7.08	0.67
C10:0	-	-	0.78
C12:0	1.55	0.66	12.97
C14:0	2.21	5.14	5.18
C16:0	21.20	28.28	10.95
C16:1 *cis 9*	-	-	0.10
C17:0	-	-	0.08
C18:0	4.13	4.18	2.79
C18:1 *cis 9*	3.64	8.23	26.65
C18:1 *cis 11*	-	-	0.73
C18:2 *cis 9*, *12*	19.05	15.48	36.16
C20:0	1.80	5.96	0.43
C18:3 *cis 9*, *12*, *15*	37.30	10.03	2.01
C22:0	0.88	9.91	0.21
C24:0	0.41	5.06	0.29
SFA	34.92	63.69	34.35
USFA	65.08	36.31	65.65
MUFA	3.95	8.85	27.48
PUFA	61.13	27.46	38.17

^1^ ME = Metabolism energy; SFA = saturated fatty acid; USFA = unsaturated fatty acid; MUFA = monounsaturated fatty acid; PUFA = polounsaturated fatty acid.

**Table 2:** Effect of dietary treatments on growth performance of lambs.

Performance Measurements	C	T1	T2	T3	T4	SEM	*p*-Value
BW gain, kg	19.9 ^b^	22.0 ^a^	21.5 ^a^	17.4 ^b^	20.5 ^b^	1.72	0.07
Total DMI, kg	145.7 ^b^	155.5 ^a^	154.6 ^a^	133.2 ^b^	146.3 ^b^	9.71	0.005
FCR	8.05	7.12	7.29	7.93	7.15	0.25	0.18

^a,b^ Within a row, means without a common superscript were significantly different. C = total mixed ration (TMR) control; T1 = TMR + regular alfalfa hay; T2 = TMR + 1 cm particle chopped alfalfa hay; T3 = TMR+ regular wheat straw; T4 = TMR + 1 cm particle length chopped wheat straw. BW gain = body weight gain; DMI = dry matter intake; FCR = feed conversion ratio.

**Table 3:** The effect of dietary treatment on ruminal volatile fatty acid concentrations (mol%).

VFA	Acetic	Propionic	Butyric	Acetic: Propionic
C (mol%) %	15.94 ^b^ 0.45	14.57 ^a^ 0.39	63.3 ^a^ 0.17	1.09 ^c^
T1 (mol%) %	16.79 ^a^ 0.47	13.14 ^a^ 0.37	55.2 ^a,b^ 0.16	1.29 ^b^
T2 (mol%) %	17.28 ^a^ 0.50	11.04 ^b^ 0.32	59.01 ^a^ 0.17	1.58 ^a^
T3 (mol%) %	17.48 ^a^ 0.48	13.90 ^a^ 0.38	47.78 ^b^ 0.13	1.27 ^b^
T4 (mol%) %	17.53 ^a^ 0.50	12.61 ^a^ 0.36	51.55 ^b^ 0.15	1.39 ^b^
SEM	1.93	1.56	5.16	0.05
*p*-value	0.09	0.40	0.01	0.04

^a,b,c^ Within a column, means without a common superscript are significantly different. C = TMR; T1 = TMR + regular alfalfa; T2 = TMR + 1 cm particle size alfalfa; T3 = TMR + regular wheat straw; T4 = TMR + 1 cm particle size wheat straw.

**Table 4:** Fatty acid profile of fat longissimus dorsi muscle fat from Naemi lambs (n = 4 per dietary group) fed different experimental diets.

Diets							
Fatty Acid (g/100 g)	C	T1	T2	T3	T4	SE	*p*-Value
C10:0	0.16	0.19	0.12	0.17	0.13	0.02	0.33
C11:0	0.08	0.12	0.05	0.07	0.04	0.03	0.81
C12:0	0.40	0.39	0.30	0.37	0.53	0.09	0.66
C13:0	0.10	0.09	0.06	0.08	0.03	0.03	0.82
C14:0 *iso*	-	0.06	0.04	0.04	0.04	0.01	0.75
C14:0	5.17	4.86	4.71	5.07	5.28	0.61	0.74
C15:0 *antiso*	0.06 ^b^	0.13 ^ab^	0.10 ^ab^	0.13 ^ab^	0.16 ^a^	0.02	0.04
C15:0 *iso*	0.36	0.28	0.26	0.31	0.30	0.04	0.52
C14:1 *cis9*	0.13	0.14	0.16	0.18	0.12	0.02	0.49
C15:0	0.98	0.89	0.80	0.90	0.68	0.11	0.52
C16:1 *cis7*	0.13	0.15	0.09	0.11	0.13	0.05	0.79
C16:0 *iso*	0.16	0.20	0.18	0.19	0.18	0.02	0.81
C17:0 *iso*	0.24	0.17	0.14	0.20	0.15	0.04	0.35
C16:0	25.57	26.73	26.12	26.31	24.70	1.21	0.85
C17:0 *antiso*	1.80	1.53	1.39	1.71	1.26	0.23	0.55
C17:0	2.70	2.55	2.50	2.74	2.33	0.24	0.82
C16:1*cis 9*	1.32	1.93	1.71	1.88	1.35	0.18	0.15
C18:0 *iso*	0.12	0.15	0.14	0.15	0.12	0.02	0.46
C17:1 *cis10*	0.90	1.16	0.97	1.27	0.71	0.25	0.64
C18:0	14.75	15.31	15.21	14.05	19.57	1.72	0.39
C18:1 *trans9*	0.52 ^a^	0.41 ^ab^	0.30 ^ab^	0.16 ^b^	0.19 ^b^	0.08	0.04
C19:0	7.90 ^a^	1.94 ^b^	2.86 ^b^	3.39 ^b^	4.31 ^ab^	1.23	0.04
C18:1 *cis9*	30.74	34.90	35.67	34.81	30.82	1.72	0.22
C18:1*cis11*	1.17	1.09	0.99	1.25	1.14	0.10	0.54
C18:1 *cis13*	0.29 ^b^	0.48 ^ab^	0.56 ^a^	0.39 ^ab^	0.51 ^ab^	0.07	0.02
C19:0 *iso*	0.13	0.14	0.12	0.12	0.20	0.02	0.31
C18:2 *trans 9*,*12*	0.18 ^c^	0.29 ^b^	0.33 ^a^	0.28 ^bc^	0.21 ^bc^	0.03	0.04
C18:2 *trans 12*, *15*	0.08	0.13	0.15	0.15	0.11	0.03	0.64
C18:2 *cis9 trans12*	3.01 ^ab^	2.33 ^c^	3.33 ^a^	2.41 ^b^	3.62 ^a^	0.24	0.02
C20:2 *trans 11*,*13*	0.07	0.13	0.11	0.12	0.11	0.03	0.75
C20:0	0.10	0.11	0.11	0.11	0.13	0.01	0.92
C18:3 *cis9*,*12*,*15*	0.23 ^c^	0.45 ^a^	0.47 ^a^	0.31 ^b^	0.37 ^ab^	0.03	0.005
C18:2 *cis9 trans11*	0.34	0.60	0.56	0.51	0.53	0.08	0.30
C22:0	-	0.07	-	0.02	-	-	-
C20:4 *cis5*,*8*,*11*,*4*	0.04	0.03	0.03	0.05	0.07	0.01	0.33
C22:5 *cis7*,*10*,*13*,*16*,*19*	0.02	0.06	0.02	0.04	0.03	0.01	0.41

Means within a row with different superscripts are significantly different (*p* < 0.05), C = TMR; T1 = TMR + regular alfalfa; T2 = TMR + 1 cm particle size alfalfa; T3 = TMR + regular wheat straw; T4 = TMR + 1 cm particle size wheat straw.

**Table 5:** Fatty acid classes and indices (%) of five different experimental diets for longissimus dorsi muscle fat of Naemi lambs (n = 5 per dietary groups; values are means ± SD).

Parameter	Diet					SEM	*p*-Value
	C	T1	T2	T3	T4		
SFA	60.78	55.80	54.55	56.12	60.05	1.95	0.20
USFA	39.22	44.19	45.45	43.88	39.95	1.95	0.20
MUSFA	35.21	40.17	40.42	40.02	34.86	2.01	0.22
PUSFA	4.01 ^a,b^	4.03 ^a,b^	5.03 ^a,b^	3.86 ^b^	5.10 ^a^	0.36	0.04
n3	0.36 ^b^	0.62 ^a^	0.64 ^a^	0.50 ^a,b^	0.55 ^a,b^	0.07	0.02
n6	3.29 ^a,b^	2.77 ^b^	3.80 ^a^	2.84 ^b^	3.97 ^a^	0.26	0.04
n3/n6	9.21 ^a^	4.48 ^c^	6.27 ^b^	5.84 ^b,c^	7.19 ^a,b^	0.67	0.01
Δ9C18	69.18	70.66	71.18	70.14	62.57	3.09	0.40
AI	1.19	1.08	0.95	1.09	1.16	0.12	0.70
TI	1.39	1.55	1.37	1.43	1.59	0.13	0.77
hFA	34.75 ^b^	38.92 ^a,b^	40.70 ^a^	38.66 ^a,b^	35.92 ^a,b^	1.78	0.02
HFA	31.15	31.97	30.59	31.75	30.51	1.74	0.97
h/H	1.12	1.23	1.33	1.25	1.18	0.11	0.76

Means within a row with different superscripts are significantly different (*p* < 0.05); SFA, saturated fatty acids; MUFA, monounsaturated fatty acids; PUFA, polyunsaturated fatty acid; AI, atherogenicity index = (12:0 + 4 × 14:0 + 16:0)/(MUFA + PUFA) calculated according to Ulbricht and Southgate (1991); TI, thrombogenicity index = (12:0 + 16:0 + 18:0)/[(0.5 × MUFA) + (0.5 × n-6 PUFA) + (3 × n-3 PUFA)+ (n-3 PUFA/n-6 PUFA)] calculated according to Ulbricht and Southgate (1991); HFA, hypercholesterolemic FA (sum of C12:0, C14:0, and C16:0); hFA, hypocholesterolemic FA (C18:1 + polyunsaturated FA) h/H, hypocholesterolemic/hypercholesterolemic ratio = (C18:1 + PUFA)/(C14:0 + C16:0); desaturase activity indices were calculated according to Juárez et al. (2008) with the Δ9DS(C18) = 100 × [ C18:1/(C18:0 + C18:1)]. C = TMR; T1 = TMR + regular alfalfa; T2 = TMR + 1 cm particle size alfalfa; T3 = TMR + regular wheat straw; T4 = TMR + 1 cm particle size wheat straw.
